# Treating chronic pain with low dose ketamine and adjunct therapies within a biopsychosocial approach: a case series

**DOI:** 10.3389/fpain.2025.1675821

**Published:** 2026-01-20

**Authors:** Shahar Almog, Michelle Weiner, Jessica N. Howarth, Jenelle Becerra, Jacobo D. Fux, Meredith S. Berry

**Affiliations:** 1 Department of Health Education and Behavior, University of Florida, Gainesville, FL, United States; 2 NeuroPain Health, Hollywood, FL, United States; 3 Department of Psychology, University of Florida, Gainesville, FL, United States

**Keywords:** ketamine, chronic pain, biopsychosocial approach, ketamine assisted psychotherapy, rehabilitation, case report, case series

## Abstract

Chronic pain is an individual experience with physical and psychological dimensions. Ketamine is used in sub-anesthetic doses to treat chronic pain. We describe a proposed multidisciplinary approach with combined treatment of low-dose ketamine and pain-focused psychological and somatic therapies to benefit quality of life of disabled chronic pain patients. Beyond pain reduction, within the biopsychosocial approach, the treatment aims to achieve reduced suffering and improved pain management, functionality, and quality of life. Adopting a multidisciplinary approach can minimize exposure to ketamine and maintain a conservative ketamine dosing regimen. In this way, ketamine is not only used for the analgesic effects, but also to facilitate internal psychological processes of body-mind integration related to the pain identity and trauma. We illustrate the presented treatment approach with three cases of patients treated in a private clinic in Florida, United States. We describe the patients' original injury, ketamine and adjunct psychological and somatic therapies regimen, and short and longer-term outcomes from the patient's perspective. These results are preliminary, require replication with validated measures, and represent an opportunity for additional research and hypothesis formation. More clinical research on ketamine and adjunct therapies for chronic pain conditions is warranted to advance treatment options.

## 1 Introduction

Chronic pain, defined as pain that lasts for three or more months, affects more than 50 million (∼20%) adults in the United States (1). Pain is often comorbid with poor mental health, and approximately 12 million people experience co-occurring depression and/or anxiety symptoms (2). Pain that persists without an apparent acute injury is a complex individual experience with aspects of physical pain and emotional suffering. Thoughts, perceptions, emotions, context, and sensory inputs from the body define the pain experience, both amplifying and reducing it. Limiting beliefs, pain catastrophizing, habitual patterns of behavior, and rigid narratives reinforce pain signaling and/or disproportionate responses to incoming sensory information. These chronic maladaptive thoughts and responses result in alterations in brain circuits and interconnectivity between brain regions [for a review of the effects of chronic pain on structural and functional maladaptive neural circuitry see (3)]. These complex responses translate to a multidimensional individual experience of pain (3) with biological, psychological, and social contributions [i.e., the biopsychosocial model of pain (4)].

Although different pharmacological (e.g., opioids) and/or interventional (e.g., nerve block injections) therapeutic options are available (with varying levels of associated risks), chronic pain is difficult to treat. Multidisciplinary pain management, with psychological and physical therapies, is recommended by experts (5, 6). The biopsychosocial model of pain offers a holistic approach to pain management—aiming to reduce pain levels and teach the patient to take active control of the pain response and suffering, and improve functionality and overall quality of life despite some level of acceptable pain (5). Despite treatment advances, high rates of chronic pain persist, requiring therapeutic approaches that can address varied aspects of the chronic pain experience. The use of dissociative/psychedelic compounds (e.g., ketamine, psilocybin) for pain management is an emerging field of interest for clinicians and researchers (7, 8).

Ketamine is a dissociative anesthetic with analgesic properties. Ketamine, in sub-anesthetic doses has been increasingly used to treat a variety of diagnoses including major depressive disorder, other psychiatric conditions, and chronic pain (9). While ketamine is mainly referred to as an N-methyl-D-aspartate (NMDA) receptor antagonist, research suggests that ketamine, its enantiomers, and its metabolites have multi-receptor and more complex pharmacology than initially suggested (10). Ketamine, through the glutamate system, may trigger neural plasticity that counteracts the pathological neurocircuitry in the brain (11). Beyond the glutamate system, which contributes to ketamine's antidepressant and mood modulating effects, ketamine also targets the opioid system, which is related to the analgesic effects, and plays a role in monoaminergic systems, involved in anti-inflammatory processes (12–14). These suggested mechanisms are not fully understood but are all relevant for analgesia and potentially involved in promoting symptom relief.

Although ketamine seems to be effective for treating both chronic pain (15, 16) and mental health conditions (17), dosing protocols (e.g., dosage, duration of administration) across conditions are typically different. Ketamine intravenous (IV) dosage for depression is commonly initiated at 0.5 mg/kg over 40–50 min (18). Cumulative ketamine doses for pain conditions are commonly larger and administered over a longer timeframe. Ketamine can be administered continuously as in-patient treatment (up to 100 h) or intermittently in outpatient clinical practice. Experts recommend infusion dosage of 0.5–0.9 mg/kg/d for 4 days every 3 months (19), and IV administrations of cumulative doses of >400 mg may produce larger reductions in pain (16). Although the literature is limited, some evidence suggests that the analgesic effects can last up to three months, however, improvement in functionality appears to be limited (20).

One proposed mechanism for ketamine's effect on chronic pain is that ketamine might alleviate chronic pain by altering the affective-motivational component of pain (21). In an outpatient sample of 329 patients treated with different protocols of repeated intravenous or subcutaneous ketamine doses for pain, depression relief (not cumulative ketamine dose or anxiety) mediated the effect of ketamine therapy on reduced pain (22). Psychotherapy delivered before, or during, or right after the ketamine is suggested to enhance and prolong the benefits of the treatment for different psychiatric conditions (23). In pain patients, some case reports showed beneficial effects of ketamine and psychotherapy administered during a 5-day continuous ketamine infusion (24, 25). Together, ketamine may disrupt chronic pain response circuits and, coupled with adjuvant psychotherapies, offer an opportunity for the formation of new adaptive circuits.

The increasing use of ketamine for pain conditions and the scarcity of long-term research raise concerns about the risks associated with ketamine, and especially with higher doses. Among recreational ketamine users who use significantly higher doses, harmful effects are reported (e.g., addiction, cystitis, memory problems) and are often dose-dependent (26). While dosing protocols for mental health appear to be relatively safe (27), experts call for caution and close monitoring of pain patients (20), and some have suggested avoiding high doses of ketamine (28). In contrast to the general conception that mental health doses may be insufficient to achieve pain relief, here, we present and discuss the potential to minimize ketamine dosage for chronic pain treatment when integrated within a biopsychosocial approach in a multidisciplinary intervention. The proposed intervention with pain-focused psychological and somatic therapies is described herein, and illustrated by the cases of three individuals with chronic pain and disability who were treated in a private clinic.

### 1.1 Multidisciplinary intervention

The multidisciplinary intervention involves ketamine administration and adjunct coaching/counseling, including psychological and somatic modalities. Ketamine is administered by a physician trained in physical medicine and rehabilitation and interventional pain management, trained by the Ketamine Training Center (an organization that provides training in ketamine assisted psychotherapy). In general, the initial ketamine dosage is based on weight (0.5 mg/kg), as well as the patient's level of anxiety, medical conditions, and treatment goals. For example, high levels of anxiety related to dissociation dictate lower doses than 0.5 mg/kg. Ketamine dosage increases each session until an appropriate therapeutic dose is achieved. The therapeutic dose is defined as the ability to “surrender” to the dissociation yet recall the experience, while still gaining pain relief.

Ketamine is administered intravenously [100% bioavailable (13)], intramuscularly (IM) [90%–95% bioavailable (13)] divided into two injections separated by 12–15 min, or sublingually (SL) [20%–30% bioavailable (13)]. Per the presented approach, IV is more appropriate for physical pain relief given the ability for longer duration and dose stability. IM is often used for emotional pain facilitated by a coach or therapist, allowing for a softening of psychological defenses, resulting in deeper self-reflection and psychological insights. If the ketamine dissociation is not tolerated, ketamine is administered with a SL lozenge during a coaching session. Safety measures during ketamine administration include monitoring blood pressure and heart rate (IV, IM, SL), and oxygen saturation (IV, IM).

Psychological and somatic therapies are delivered in 60–90 min coaching sessions, conducted as integration sessions within 72 h following the ketamine administration (29). The sessions are conducted in-person or via Zoom, based on patient preferences. The psychological coaching sessions are conducted by integration coaches (i.e., Master's level Certified Coach). Coaching sessions consist of a combination of methods, including Somatic Tracking within Pain Reprocessing therapy (30), Internal Family Systems therapy (31), Eye Movement Desensitization and Reprocessing [EMDR (32)], breathwork, and mindfulness meditation. The somatic coaching is conducted by a physical therapist during the integration sessions. These sessions focus on improving functional movement by utilizing somatic tracking, nervous system regulation, and personalized mobility exercises.

## 2 Case description

To illustrate the multidisciplinary intervention we describe three cases of patients suffering from chronic pain following physical injuries, who were treated in NeuroPain Health during March 2024 to July 2024, and were followed for approximately one year after the initial treatment. The three patients received low doses of ketamine using either intravenous, intramuscular, or sublingual routes of administration in conjunction with pain-focused psychological and somatic therapies. Aligned with the biopsychosocial model of pain, beyond pain reduction, the aim of the treatment was to improve overall functionality, well-being, and quality of life and reduce psychological suffering associated with the pain experience.

Assessments included a numeric rating scale of pain (from 0 to 10), assessed at baseline and at the end of the short-term treatment phase (following one visit per week for 4–6 weeks). We collected qualitative reports regarding reduction in suffering, improvement in functionality and quality of life, and active participation in pain management (e.g., state of mind, interpretation of pain), summarized at the end of the acute treatment phase. Longer-term experiences were also collected with subjective qualitative reports. Table 1 presents the patients' characteristics, age at baseline, age at injury, type of injury/disability, comorbidities, and prior treatments. Table 2 presents the treatment protocol of the three patients. No serious adverse events occurred during ketamine administration sessions. All three patients also received physical therapy in other physical therapy clinics. Treatment outcomes, including longer-term, are summarized in Table 3. Each patient provided written consent to present their case in this report.

**Table 1:** Patient demographic and clinical characteristics and timeline.

Patient	Age at first session, Sex	Age at injury	Type of injury/disability	Comorbidities/functionality	Prior treatments
A	37 years, Male	26	Tethered spinal cord, secondary to a motor vehicle accident	Physical disability	Up to 140 mg of oxymorphone per day, surgeries
B	57 years, Female	51	Lower limb dystonia	GAD	PT, mental health counseling, lower extremity botulinum toxin injections
C	58 years, Male	52	Facial and C6–7 fractures, traumatic spinal cord injury and quadriplegia following a cycling accident, chronic facial pain	GAD	Gabapentin, Lyrica, muscle relaxants, PT

PT, physical therapy; GAD, general anxiety disorder.

**Table 2:** Intervention structure: initial loading sessions and long-term treatment.

Patient	Route of administration	Initial dose	Therapeutic/Maximal dose	Psychotherapy/Somatic therapy	Initial treatment: Number of ketamine sessions, frequency	Long-term ketamine treatment: Route, dose, frequency, other therapy
A	IM	50 mg total: 2 injections (20/30 mg)	145 mg total: 2 injections (70/75 mg)	EMDR, IFS, Pain Reprocessing, functional movement	6, 1 weekly	IM, 75–145 mg every 2–4 months
B	IV	60 mg over 60 min	135 mg over 90 min	Somatic Tracking, Pain Reprocessing, IFS, breathwork, meditation, functional movement	4, 1 weekly	SL 25 mg during KAP, 2–3 sessions every several months
C	IM	50 mg total: 2 injections (20/30 mg)	65 mg total: 2 injections (30/35 mg)	Somatic Tracking, Pain Reprocessing, IFS, breathwork, meditation, functional movement	6, 1 weekly	None, recently resumed counseling

IM, intramuscular; IV, intravenous; SL, sublingual (lozenge); EMDR, eye movement desensitization and reprocessing; IFS, internal family systems; PT, physiotherapy; KAP, ketamine assisted psychotherapy.

**Table 3:** Treatment outcomes based on the biopsychosocial model of pain.

Patient	Reductions in pain, % reduction in suffering	Taking active control in pain management	Change in functionality and quality of life—short term	Longer-term experiences
A	8/10–5/10	Reported increased ability to interpret bodily sensations.	Reported improved gait and reduced assisted walking. Began exercising, with the goal of returning to the workforce.	Reported continuing to improve gait, coordination, and muscle control. More awareness to left leg, better interpretation of pain and sensations. Reduced frequency, intensity, and duration of brain fog and pain flares. Taking in-person courses. Reduced medication doses.
	75%			
B	8/10–4/10	Reported dissociating identity from diagnosis of dystonia, and viewing diagnosis from a different perspective.	Reported improved participation in activities of daily living. Regained mobility, began swimming and cycling, resumed hobbies. Reported feeling joy in everyday life.	Reported being able to participate in daily activities, returning to work. Walking more frequently without assistance, able to swim with leg function, transitioned to riding outdoors with e-bike. Resumed hobbies and reported feelings of peace, contentment, and fun.
	50%			
C	6/10–5/10	Reported better perception of the pain, and ability to shift focus from pain to gait mechanics.	Reported 70%–80% “back” to prior level of function, resumed cycling and increased distance per ride.	Reported continuing to cycle, better coping with triggers, and with facial pain.
	10%–15%			

### 2.1 Patient A

#### 2.1.1 Case presentation

Patient A is a 37-year-old male with a history of tethered spinal cord secondary to a motor vehicle accident at age 26. His injury resulted in autonomic dysfunction, motor control deficits due to impaired proprioception, and pain that required him to medically retire as an Air Force pilot. At one point, he was prescribed up to 140 mg of oxymorphone per day for pain management. He underwent seven surgeries between 2016 and 2021 and experienced several complications including hydrocephalus and frequent cerebrospinal fluid leaks. His surgeon required him to wean off pain medications prior to his last two surgeries, after which he experienced sufficient pain relief to begin to move forward with healing and was eventually cleared to start ketamine therapy.

#### 2.1.2 Treatment

Patient A underwent six weekly IM ketamine sessions. The total initial ketamine dose given was 50 mg (two injections of 20 mg and 30 mg). Each session, the dose was increased until reaching his therapeutic total dose of 145 mg (70 mg and 75 mg). He received adjunctive psychotherapies including EMDR, Internal Family Systems, and Pain Reprocessing therapy. He also received functional movement sessions, including electrical stimulation, gait training, and balance exercises. Following the initial treatment, his long-term treatment includes an IM ketamine session with coaching every two to four months, as needed.

#### 2.1.3 Outcomes and follow-up

Patient A reported positive changes in pain and level of function since undergoing ketamine combined with mental health and functional mobility coaching, and PT. His average pain decreased from 8/10 to 5/10. Overall, he reported a 75% reduction in his suffering that he attributed to the combination of ketamine sessions followed by adjuvant therapies for integration. Before ketamine and adjunctive therapies, he spent time physically “curled up” and mentally in “fight-or-flight” mode. Post-treatment, he reported improved muscular flexibility and being able to perform a push-up for the first time since his injury. Before ketamine and the adjunctive therapies, he ambulated with a single-point cane or rolling walker and would frequently drag his left leg. Post treatment, he typically ambulates indoors without an assistive device and ambulates outdoors or when fatigued with a single-point cane. He reported improved proprioception and a more accurate ability to interpret sensations in his body. He has a goal of returning to the workforce, which he said was not an option before. With long-term booster treatments, Patient A reported continued improvements, reduced pain flares, better interpretation of pain and sensations, and reduced use of other medications (i.e., 50% reduction in Gabapentin dose, 80% reduction in Cymbalta dose).

### 2.2 Patient B

#### 2.2.1 Case presentation

Patient B is a 57-year-old female who was diagnosed with lower limb dystonia at age 51. Before symptoms began, she retired and moved into her dream home in the Florida Keys. She was very active, competed in triathlons, and enjoyed diving. Symptoms started as mild toe clawing with running, and over five years progressed to a point where she had to use a knee scooter or wheelchair for mobility. All forms of exercise would exacerbate her symptoms. As a result she became inactive. She tried PT and mental health counseling with no significant improvement. Lower extremity botulinum toxin injections (400 units every 90 days) provided some relief, allowing her to ambulate short distances and provided a 50% reduction in pain. Her physical symptoms were improving but she began to experience uncontrolled anxiety and panic attacks.

#### 2.2.2 Treatment

Patient B completed four weekly IV ketamine sessions, initially dosed at 1 mg/kg (60 mg total) over one hour and progressed to her therapeutic dose of 135 mg over 90 min per session. Due to anxiety levels, the treatment shifted from high-dose IV to low-dose 25 mg SL lozenge during adjuvant psycholytic therapies that included somatic tracking and Pain Reprocessing, Internal Family Systems, breathwork, and meditation, as well as functional movement counseling. Long-term, based on her needs and preferences, Patient B receives an occasional IV followed by 2–3 SL during coaching sessions every several months, as needed.

#### 2.2.3 Outcomes and follow-up

Before ketamine and adjuvant therapies, Patient B reported daily pain of 8/10. She reported that ketamine and adjuvant therapies have “dialed down” her pain to 4/10 and reduced her suffering by 50%, allowing her to “feel joy in everyday life again”. During one of the IV ketamine sessions patient B had the realization that “the power to heal is within me”. Through Internal Family Systems work, she reported she was able to recognize traumatic events that preceded her diagnosis, process the grief of repetitive losses she experienced (e.g., loss of husband), and begin to heal. She weaned off botulinum toxin (100 units every 90 days, due to negative impact on swallowing and respiration) and reported improved participation in activities of daily living. Long-term, she gradually returned to swimming and cycling. She reported that ketamine allowed her to disassociate her identity from her diagnosis of dystonia, and that mental health counseling has allowed her to view her diagnosis from a different perspective. With the combined therapies, she regained mobility and returned to hobbies, which significantly improved her quality of life.

### 2.3 Patient C

#### 2.3.1 Case presentation

Patient C is a 58-year-old male who sustained severe facial fractures and a C6–7 fracture that resulted in a traumatic spinal cord injury and quadriplegia following a cycling accident in the Swiss Alps at age 52. He underwent facial reconstruction and cervical spinal fusion in Switzerland. Once medically stable, he flew to the United States and participated in inpatient and outpatient rehabilitation. He regained significant function, including the ability to walk without an assistive device. He returned to cycling despite mild impairments in lower extremity sensation including proprioception and upper extremity strength. Continued facial pain was unresponsive to Gabapentin, Lyrica, and muscle relaxants.

#### 2.3.2 Treatment

Patient C completed six IM ketamine sessions with an initial dosage of 50 mg (20 mg and 30 mg). By his third session, his dose progressed to 30 mg and 35 mg per session, which became his therapeutic dose. He also participated in therapy sessions that included somatic tracking and Pain Reprocessing, Internal Family Systems, breathwork, meditation, and functional movement. Patient C did not continue to receive ketamine long-term. Recently, he resumed psychotherapy sessions in the clinic.

#### 2.3.3 Outcomes and follow-up

Patient C reported reductions in average pain from 6/10 pre-treatment to 5/10, and 10%–15% reduction in suffering post-treatment, with “better” perceptions about the pain. He reported the regimen helped him address trauma, resulting in improved mental health. He reported he was walking “better”. He attributes this to his ability to shift his focus from the pain to his gait mechanics. Additionally, he continued cycling and increased the distance up to 50–70 miles per ride. He reported feeling like he was 70%–80% “back” to his prior level of function. Nine months following his last ketamine treatment in the clinic, Patient C reported enhanced awareness of triggers, which helps him better avoid or mitigate them, and acceptance of the physical change of his face after his injury. He acknowledged that the pain-focused psychotherapy was most beneficial, and although his facial pain has only slightly improved, he felt he could tolerate it better.

## 3 Patient perspective

The three patients described changes related to mental and physical aspects of pain and quality of life. For example, Patient A summarized the treatment as “It has and continues to be nothing short of life saving. …A catalyst for healthy lifestyle changes that have helped improve my quality of life. …The way this has helped with mind/body connection and pain (outside the acute effects during the session) has been completely counterintuitive to every other Western approach I have tried and the only one that has actually allowed the space for my body/mind to begin healing”.

## 4 Discussion

Chronic pain is a highly complex individual experience. We propose a multidisciplinary intervention within a biopsychosocial approach, combining sub-anesthetic ketamine and pain-focused therapies. We reported on three cases to illustrate the treatment and outcomes. The three patients suffering from chronic pain for several years received individualized combined ketamine and supporting therapies. After one to two months, the three patients achieved reduced pain levels with improvements in functionality and quality of life. Improvements continued for longer periods with (Patients A and B) and without (Patient C) ketamine maintenance treatment, tailored to patient preferences and needs. These preliminary findings add to the growing body of literature suggesting ketamine could be an impactful tool to facilitate internal processes of body-mind integration when treating pain within a multidisciplinary approach.

Adjuvant therapies can serve as a focused integration of the ketamine dissociation experience to maximize the therapeutic potential of neuroplasticity (11). Ketamine might facilitate internal processes related to pain and traumatic injury by allowing calm access to trauma, self-insight, and emotion regulation (33). Integration practitioners and therapists should help patients with chronic pain recognize the absence of immediate physical danger, teach nervous system regulation tools, and allow a recalibration of response to perceived dangers. This will allow for improved pain management (e.g., interpretation of sensations and pain) and functional mobility. The combination of ketamine-induced neuroplasticity (11) and adjuvant therapies may simultaneously contribute to dissociating existing pain circuits and developing new adaptive thoughts and responses. The combined therapies can target chronic pain and any comorbid mental health conditions (e.g., anxiety) caused by a change in one's physical function, as experienced by the three patients presented. Ketamine is involved in multiple systems promoting different processes related to analgesia, pain pathways modulation, inflammatory processes, and neuroplasticity (12–14). Each of these suggested mechanisms may underlie the holistic benefits and longer-term functional outcomes of the ketamine assisted multidisciplinary treatment. These may include reduced pain and improved affective response, pain interpretation, pain reactivity, mood, and overall functionality in life. Nevertheless, the exact mechanism(s) that underlie the observed outcomes are still undetermined and should be studied further in future research.

In the presented cases, the multidisciplinary approach utilizing multiple therapies produced reductions in pain with relatively low doses of ketamine, compared to typical dosing protocols for pain. While ketamine is a promising treatment for many conditions, it is also associated with physical and addictive risks. These issues may be exacerbated by longer-term ketamine treatments, although longer-term comprehensive research and follow-up are lacking. Anecdotal evidence suggests similarities in toxicity symptoms (e.g., gastrointestinal tract toxicity “K-cramps”, urotoxicity cystitis) among chronic pain patients treated with higher doses of ketamine and recreational ketamine users (28). A multidisciplinary approach that leverages low-dose ketamine may improve pain and quality of life while minimizing ketamine exposure, reducing associated risks, and enhancing long-term safety. Systematic research with longer-term follow-up is needed to develop specific protocols for different types of chronic pain, with optimal ketamine doses, and most effective psychotherapies.

Other strengths of the proposed intervention include the flexibility in the ketamine dosing protocol tailored to the patient's needs, tolerance, and preferences. The purpose of the dosing regimen is to reach a dissociative state where self-insights related to pain (e.g., to pain identity, the mental and emotional stage of the acute pain transforming into chronic) can be elicited. Dividing the dose into two injections as in the present case series allows the medical team to check in with the patient and adjust dosage before the second IM injection. Critically, this approach can facilitate relatively lower and shorter doses than typically utilized for chronic pain (19) while still achieving beneficial outcomes.

Although beneficial, the multidisciplinary approach requires several providers on the treatment team and can present cost and accessibility limitations. When all services are in-house (as in the present report), this allows for efficient coordination in scheduling and treatment progression. It may be optimal to establish a team of professionals prior to initiation of the treatment, or, alternatively, establish open communication with the providers who may already be working with the patient.

The present report has several limitations. First, the three cases that were chosen to illustrate the multidisciplinary approach are broadly representative of commonly observed pain-disabled patients within the clinic. Despite the ecologically valid and broad representation, the risk of selection bias limits the generalization of these cases without further evidence from systematic investigations and clinical studies. It is also important to acknowledge that ketamine may not be beneficial for everyone, and some patients do not benefit from the presented treatment approach. Future research should strive to identify those who respond well to integrate science and practice, and allow practitioners to predict and tailor treatment to those likely to benefit. Second, patients who seek ketamine assisted therapies for chronic pain commonly have a long history of engaging with different therapies and treatments in the past and/or currently. Thus, we are limited in attributing the improvements solely to the ketamine and/or other specific therapies utilized in the intervention. Moreover, the ketamine treatment was highly individualized as seen in the three presented cases (e.g., routes of administration, doses, adjunct therapies) which further limits conclusions regarding the effectiveness of each component of the intervention. The multi-receptor activity of ketamine adds to the complexity and complicates interpretation, especially regarding improved pain outcomes (e.g., opioid receptor activity). Lastly, we presented only self-reported data and did not collect validated measures such as functionality, pain catastrophizing, or pain interference to capture pain or disability improvements. Acknowledging these limitations, our report seeks to contribute to the evolving field of ketamine-based and psychedelic-inspired approaches to pain management, to generate hypotheses for future research, and to call for rigorous clinical studies with validated and objective outcomes to improve chronic pain treatment protocols.

In conclusion, low-dose ketamine and adjuvant pain-focused therapies can be combined in a multidimensional treatment protocol based on a biopsychosocial approach to treat chronic pain. In the three cases presented to illustrate the intervention, the treatment achieved some level of pain relief, reduced emotional suffering, and improved active management of pain, functionality, and quality of life. Ketamine may help the individual separate or dissociate from the pain identity and induce neuroplasticity necessary for healing and building a new belief system around the pain. Combined protocols of ketamine and supporting psychological and somatic therapies could utilize lower doses of ketamine than are commonly prescribed for chronic pain to minimize risks associated with ketamine. More systematic research is needed on combined ketamine and adjuvant therapies for chronic pain.

## Data Availability

The original contributions presented in the study are included in the article/Supplementary Material, further inquiries can be directed to the corresponding authors.

## References

[1] Rikard SM. Chronic pain among adults—United States, 2019–2021. MMWR Morb Mortal Wkly Rep. (2023) 72(15):379–85. 10.15585/mmwr.mm7215a1 37053114 PMC10121254

[2] De La Rosa JS Brady BR Ibrahim MM Herder KE Wallace JS Padilla AR Co-occurrence of chronic pain and anxiety/depression symptoms in U.S. adults: prevalence, functional impacts, and opportunities. Pain. (2024) 165(3):666–73. 10.1097/j.pain.0000000000003056 37733475 PMC10859853

[3] Kuner R Kuner T. Cellular circuits in the brain and their modulation in acute and chronic pain. Physiol Rev. (2021) 101(1):213–58. 10.1152/physrev.00040.2019 32525759

[4] Gatchel RJ. Comorbidity of chronic pain and mental health disorders: the biopsychosocial perspective. Am Psychol. (2004) 59(8):795–805. 10.1037/0003-066X.59.8.795 15554853

[5] Hylands-White N Duarte RV Raphael JH. An overview of treatment approaches for chronic pain management. Rheumatol Int. (2017) 37(1):29–42. 10.1007/s00296-016-3481-8 27107994

[6] Sharpe L Jones E Ashton-James CE Nicholas MK Refshauge K. Necessary components of psychological treatment in pain management programs: a Delphi study. Eur J Pain. (2020) 24(6):1160–8. 10.1002/ejp.1561 32187442

[7] Castellanos JP Woolley C Bruno KA Zeidan F Halberstadt A Furnish T. Chronic pain and psychedelics: a review and proposed mechanism of action. Reg Anesth Pain Med. (2020) 45(7):486–94. 10.1136/rapm-2020-101273 32371500

[8] Strand NH Whitney M Johnson B Dunn T Attanti S Maloney J Pain and perception: exploring psychedelics as novel therapeutic agents in chronic pain management. Curr Pain Headache Rep. (2025) 29(1):15. 10.1007/s11916-024-01353-0 39775134

[9] Nowacka A Borczyk M. Ketamine applications beyond anesthesia—a literature review. Eur J Pharmacol. (2019) 860:172547. 10.1016/j.ejphar.2019.172547 31348905

[10] Levinstein MR Budinich RC Bonaventura J Schatzberg AF Zarate CA Michaelides M. Redefining ketamine pharmacology for antidepressant action: synergistic NMDA and opioid receptor interactions? Am J Psychiatry. (2025) 182(3):247–58. 10.1176/appi.ajp.20240378 39810555 PMC11872000

[11] Aleksandrova LR Phillips AG. Neuroplasticity as a convergent mechanism of ketamine and classical psychedelics. Trends Pharmacol Sci. (2021) 42(11):929–42. 10.1016/j.tips.2021.08.003 34565579

[12] Jóźwiak-Bębenista M Sokołowska P Wiktorowska-Owczarek A Kowalczyk E Sienkiewicz M. Ketamine—a new antidepressant drug with anti-inflammatory properties. J Pharmacol Exp Ther. (2024) 388(1):134–44. 10.1124/jpet.123.001823 37977808

[13] McIntyre RS Rosenblat JD Nemeroff CB Sanacora G Murrough JW Berk M Synthesizing the evidence for ketamine and esketamine in treatment-resistant depression: an international expert opinion on the available evidence and implementation. Am J Psychiatry. (2021) 178(5):383–99. 10.1176/appi.ajp.2020.20081251 33726522 PMC9635017

[14] Wilkowska A Cubała WJ. The downstaging concept in treatment-resistant depression: spotlight on ketamine. Int J Mol Sci. (2022) 23(23):14605. 10.3390/ijms232314605 36498934 PMC9738502

[15] Corriger A Voute M Lambert C Consortium O Pereira B Pickering G. Ketamine for refractory chronic pain: a 1-year follow-up study. Pain. (2022) 163(4):690. 10.1097/j.pain.0000000000002403 34252909

[16] Orhurhu V Orhurhu MS Bhatia A Cohen SP. Ketamine infusions for chronic pain: a systematic review and meta-analysis of randomized controlled trials. Anesth Analg. (2019) 129(1):241–54. 10.1213/ANE.0000000000004185 31082965

[17] Walsh Z Mollaahmetoglu OM Rootman J Golsof S Keeler J Marsh B Ketamine for the treatment of mental health and substance use disorders: comprehensive systematic review. BJPsych Open. (2022) 8(1):e19. 10.1192/bjo.2021.1061 PMC8811778 35040425

[18] O’Brien B Wilkinson ST Mathew SJ. An update on community ketamine practices. Am J Psychiatry. (2022) 179(5):393–4. 10.1176/appi.ajp.21111086 35491568

[19] Voute M Riant T Amodéo J-M André G Barmaki M Collard O Ketamine in chronic pain: a Delphi survey. Eur J Pain. (2022) 26(4):873–87. 10.1002/ejp.1914 35092320

[20] Niesters M Martini C Dahan A. Ketamine for chronic pain: risks and benefits. Br J Clin Pharmacol. (2014) 77(2):357–67. 10.1111/bcp.12094 23432384 PMC4014022

[21] Yang Y Maher DP Cohen SP. Emerging concepts on the use of ketamine for chronic pain. Expert Rev Clin Pharmacol. (2020) 13(2):135–46. 10.1080/17512433.2020.1717947 31990596

[22] Voute M Lambert C Pereira B Pickering G. Assessment of initial depressive state and pain relief with ketamine in patients with chronic refractory pain. JAMA Netw Open. (2023) 6(5):e2314406. 10.1001/jamanetworkopen.2023.14406 37204789 PMC10199354

[23] Drozdz SJ Goel A McGarr MW Katz J Ritvo P Mattina GF Ketamine assisted psychotherapy: a systematic narrative review of the literature. J Pain Res. (2022) 15:1691–706. 10.2147/JPR.S360733 35734507 PMC9207256

[24] Keizer BM Roache JD Jones JR Kalpinski RJ Porcerelli JH Krystal JH. Continuous ketamine infusion for pain as an opportunity for psychotherapy for PTSD: a case series of ketamine-enhanced psychotherapy for PTSD and pain (KEP-P2). Psychother Psychosom. (2020) 89(5):326–9. 10.1159/000507095 32248200

[25] Ocker AC Shah NB Schwenk ES Witkowski TA Cohen MJ Viscusi ER. Ketamine and cognitive behavioral therapy for rapid opioid tapering with sustained opioid abstinence: a case report and 1-year follow-up. Pain Pract. (2020) 20(1):95–100. 10.1111/papr.12829 31408575

[26] Van Amsterdam J Van Den Brink W. Harm Related to Recreational Ketamine Use and Its Relevance for the Clinical Use of Ketamine. A Systematic Review and Comparison Study (2022). 10.1080/14740338.2021.1949454 34176409

[27] Feifel D Dadiomov D Lee KC. Safety of repeated administration of parenteral ketamine for depression. Pharmaceuticals. (2020) 13(7):151. 10.3390/ph13070151 32668686 PMC7408561

[28] Bell RF Kalso EA. Ketamine for pain management. Pain Rep. (2018) 3(5):e674. 10.1097/PR9.0000000000000674 30534625 PMC6181464

[29] Batievsky D Weiner M Kaplan SB Thase ME Maglione DN Vidot DC. Ketamine-assisted psychotherapy treatment of chronic pain and comorbid depression: a pilot study of two approaches. Front Pain Res. (2023) 4:1127863. 10.3389/fpain.2023.1127863 PMC10235727 37273242

[30] Ashar YK Gordon A Schubiner H Uipi C Knight K Anderson Z Effect of pain reprocessing therapy vs placebo and usual care for patients with chronic back pain: a randomized clinical trial. JAMA Psychiatry. (2022) 79(1):13–23. 10.1001/jamapsychiatry.2021.2669 34586357 PMC8482298

[31] Schwartz RC. Internal Family Systems Therapy. New York: Guilford (1995).

[32] Shapiro F. Efficacy of the eye movement desensitization procedure in the treatment of traumatic memories. J Trauma Stress. (1989) 2(2):199–223. 10.1002/jts.2490020207

[33] Almog S Garcia-Romeu A Walker KA Weiner M Berry MS. Self-reported improvements in comorbid post-traumatic stress disorder symptoms, depression, anxiety, and sleep among real-world patients receiving medical ketamine: exploring the role of adjunct therapies. Eur J Trauma Dissociation. (2025) 9(3):100572. 10.1016/j.ejtd.2025.100572 PMC12962596 41800353

